# Supplementary material for: A comprehensive protocol for PDMS fabrication for use in cell culture
Source: PLoS One. 2025 May 12;20(5):e0323283. doi: 10.1371/journal.pone.0323283 (PMC12068733; doi:10.1371/journal.pone.0323283)
Supplement: S1 File — (PDF) [file pone.0323283.s001.pdf]

A Comprehensive Protocol for PDMS Fabrication for use in Cell Culture

RESERVED DOI:
10.17504/protocols.io.36wgqnn7xgk5/v1

Aisling J. Greaney[1], Clíona M. McCarthy[1], Jishnu Padacherri Vethil[1], Mannthalah Abubaker[1], Erin C. Reardon[1], Frederick D. Crowley[1], Eoghan M. Cunnane[1], John J.E. Mulvihill[1]

[1]University of Limerick

John J.E. Mulvihill
University of Limerick

Protocol Info: Aisling J. Greaney, Clíona M. McCarthy, Jishnu Padacherri Vethil, Mannthalah Abubaker, Erin C. Reardon, Frederick D. Crowley, Eoghan M. Cunnane, John J.E. Mulvihill . A Comprehensive Protocol for PDMS Fabrication for use in Cell Culture. **protocols.io** **https://protocols.io/view/a-comprehensive-protocol-for-pdms-fabrication-for-desw3efe**

Created: May 30, 2024

Last Modified: February 27, 2025

Protocol Integer ID: 100918

Keywords: Silicone, PDMS, Sylgard 184, Sylgard 527, Cell culture

Funders Acknowledgements:
Irish Research Council
Grant ID: GOIPG/2021/1433

Disclaimer

None

Abstract

Cells exhibit remarkable sensitivity to the mechanical properties of their surrounding matrix, particularly stiffness changes, a phenomenon known as cellular mechanotransduction. In vivo, tissues exhibit a wide range of stiffness, from kilopascals (kPa) to megapascals (MPa), which can alter with aging and disease. Traditional cell culture methods employ plastic substrates with stiffness in the gigapascal range, which does not accurately mimic the physiological conditions of most biological tissues. Therefore, employing substrates that can be engineered to span a wide range of stiffnesses, closely resembling the native tissue environment, is crucial for obtaining results that more accurately reflect cellular responses *in vivo*.

Polydimethylsiloxane (PDMS) substrates are widely used in cell culture for their ability to simulate tissue stiffness, but their optimization presents several challenges. Fabrication requires precise control over mixing, weighing, and curing to ensure reproducible mechanical properties. Inconsistent preparation can lead to improperly cured PDMS substrates, compromising experimental outcomes. Additionally, PDMS's inherent hydrophobicity poses challenges for cell attachment, necessitating surface modifications to enhance adhesion. Moreover, the risk of contamination during the sterilization process necessitates stringent protocols to maintain cell culture integrity. These challenges are further compounded by substrate autofluorescence which can cause difficulties when imaging cells.

The aim of this study is to develop a standardized method for fabricating PDMS substrates with tuneable stiffness, ranging from kPa to MPa, suitable for diverse cell types using standard laboratory equipment. This method aims to minimize the complexity and equipment required for PDMS fabrication, ensuring reproducibility and ease of use. Achieving consistent and contaminant-free PDMS substrates will facilitate a broader adoption of these substrates in mechanobiology research and improve the relevance of *in vitro* models to *in vivo* conditions. Ultimately, contributing to a more comprehensive understanding of cellular responses to mechanical cues in health and disease.

Attachments

FILE

S3 Fig.tif

2.6MB

Materials

Oven set to 60 °C
Dow Corning Sylgard 184 Part A/B
Dow Corning Sylgard 527 Part A/B
Weighing scales
Plastic stirring rods
Pasteur pipettes
Tube rack
15ml/50ml centrifuge tubes
Weigh boats
10ml syringes
Well plates
60 mm petri dishes
15 mm tissue punch
Centrifuge for tubes and plates
70% ethanol
1X sterile PBS
Laminar flow hood with UV light
Pipetting aid
Serological pipettes
Waste beaker
Dopamine hydrochloride
10 mM Tris-HCl pH 8.5 solution
60 ml syringe
0.45 um syringe filter
Rat tail collagen type 1

Note

Other brands for consumables are available

Protocol materials

Collagen I, Rat Tail **Corning Catalog #**354236 Step 12
SYLGARD™ 184 Silicone Elastomer Kit **Dow Corning Catalog #**04019862 Step 1
SYLGARD™ 527 Silicone Dielectric Gel Kit **Dow Corning Catalog #**2270030 Step 1
Phalloidin **Proteintech Catalog #**PF00003 Step 16

Safety warnings

Wear appropriate PPE for the duration of the experiment i.e. lab coat, disposable gloves and safety glasses).

Refer to the appropriate safety data sheet for the reagents and chemicals for correct handling and storage.

Dispose of used reagents in the appropriate waste bins.

Before start

Prepare sterile water and 1X sterile PBS for washing during gel sterilization.
Prepare 10 mM Tris-HCl pH 8.5 solution.
Ensure the oven is switched on and set to 60 °C

Equipment Setup: Silicone gels

1 Gather all required materials and equipment, including silicone base (Part A) and curing agent (Part B) (SYLGARD™ 184 Silicone Elastomer Kit **Dow Corning Catalog #**04019862 and SYLGARD™ 527 Silicone Dielectric Gel Kit **Dow Corning Catalog #**2270030), weighing scales, stirring rods, Pasteur pipettes, centrifuge tubes, tube rack, weigh boats, syringes, 15mm puncher, well plates, and 60 mm dishes. Set up the equipment under a fume hood or hanging fume hood.

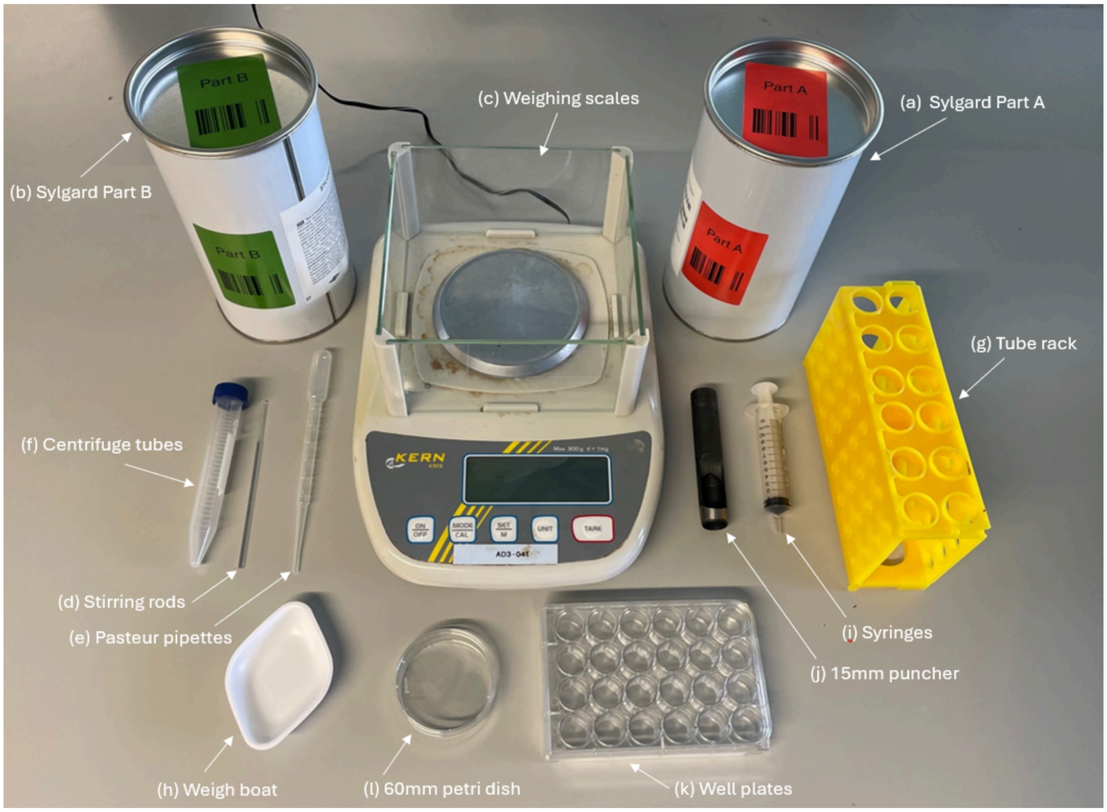

Preparation of Silicone Gels

3d 1h 35m

2 Weigh the desired amounts of base (Part A) and curing agent (Part B) in a weigh boat, ensuring precise measurements for each part.

3 Mix Sylgard 184 and Sylgard 527 using the following mixing procedures. 20m

- For Sylgard 184, stir Part A and Part B for approximately 00:06:00 , for masses less than 10 g , with a stirring rod in the weigh boat until thoroughly mixed [1,2]. Increase mixing time as necessary for larger masses. Pour the mixture into a centrifuge tube.

- For Sylgard 527, stir Part A and Part B for 00:06:00 , for masses less than 10 g , with a stirring rod in the weigh boat [3]. Transfer the mixture to a centrifuge tube, ensuring centrifuge tubes are filled only halfway to prevent overflow during mixing. Mix vigorously with a pasteur pipette for an additional 00:08:00 . Increase mixing times as necessary for larger masses.

4 Centrifuge the silicone mixtures at 600 rcf, 21°C, 00:05:00 to remove air bubbles. 5m

5 Cast the Sylgard mixtures into dishes or well plates, ensuring the same mass per well. Refer to Table 1 for recommended volumes for different well plates and petri dishes. 5m

A	B	C
**Plates / Dishes**	**Surface area (cm²)**	**Silicone mixture (g)**
35mm	8.8	1.228
60mm	21.5	3
100mm	56.7	7.912
150mm	145	20.233
6-well	9.5	1.34
12-well	3.83	0.53
24-well	1.93	0.265

Table 1 Recommended masses for silicone mixtures in various petri dishes and well plates to maintain consistent thickness.

If air bubbles form during casting, plates can be centrifuged again at 300 rcf, 21°C, 00:05:00 .

6 Transfer the silicone gels to an oven and cure at 60 °C for 24-48 hours. Sylgard 184 gels cure within 24:00:00 , however, some Sylgard 527 gels need 48:00:00 to cure. 3d 1h

- To increase the stiffness of Sylgard 184 gels, remove the gels from the petri dishes and post-cure at 200 °C for 01:00:00 (S3 Fig).

Microindentation of Silicone gels

7 Microindentation is performed using the Chiaro Nanoindenter (Optics 11, the Netherlands). As Sylgard 527 gels are adhesive after curing, coat the gels in [M] 5 % (v/v) BSA solution for 02:00:00 before indenting to prevent the probe from adhering to the gel. Sylgard 184 gels do not require coating with [M] 5 % (v/v) BSA. Before each experiment, calibrate the probe by submerging the cantilever in deionised water and using a glass calibration dish. Conduct the study at Room temperature to prevent measurement errors due to temperature drift. Add deionised water to the gels via pipetting to prevent static forces during 2h

the indentation process [4]. Perform indentations across the wells and petri dishes. The probes used to perform microindentation of the Sylgard 184 gels are within the 180 N/m cantilever stiffness and 50 μm radius spherical tip range of probes. The probes used to perform microindentation of the Sylgard 527 gels are within the 0.5 N/m cantilever stiffness and 50 μm radius spherical tip range of probes. Execute an automated 5x6 matrix scan, spaced in 1000 μm increments to avoid overlapping. Use the indentation mode with 8000 nm set as the indentation depth and 5000 nm/s as the indentation speed. This process ensures suitable microindentation of silicone gels ranging from 1 kPa to 10 MPa.

8 To perform gel thickness analysis, begin by calibrating the probe with a cantilever stiffness of 180 N/m and a spherical tip with a 50 μm radius. Set the indentation depth to 1 nm. Conduct an automated 6x6 matrix scan, with 1000 μm spacing increments, across an identical empty well plate to the one used for curing the gels. Next, perform the same matrix scan across the Sylgard 184 gels, increasing the indentation depth to 8000 nm. Ensure that the height of the indenter arm remains constant throughout this process. Subsequently, calibrate the probe with a cantilever stiffness of 0.5 N/m, maintaining the spherical tip radius at 50 μm. Execute a matrix scan, with indentation depth set to 1 nm, on the empty well plate once more. Finally, repeat the matrix scan across the Sylgard 527 gels, again increasing the indentation depth to 8000 nm.

Procedure for Sterilizing the Silicone Gels after Curing

9 For gels cured in well plates or petri dishes, spray the gels with 70% ethanol and then transfer them to a sterile hood. Then add a small amount of 70% ethanol to each well or dish which will be seeded with cells. Without removing the ethanol, sterilize the well plates and petri dishes under UV light in the sterile hood for 00:30:00 . After sterilization, remove the ethanol and wash each well and petri dish twice with sterile water. 1h

- If using post-cured gels in 24-well plates, punch out discs from the post-cured gel using a 15mm puncher. Bathe these gel discs in a small amount of 70% ethanol, transfer them to a sterile hood, and wash twice in a sterile water bath. Allow the discs to dry, then insert them into the bottom of the 24-well plates. Add 200 µL of 70% ethanol to each well, sterilize for 00:30:00 under UV light in a sterile hood, remove the ethanol, and wash twice with sterile water.

Procedure for rendering the gels hydrophilic

10 Make a [M] 0.01 % (v/v) PDA solution by dissolving 1 mg dopamine hydrochloride in 10 mL of [M] 0.01 Molarity (M) Tris HCl pH 8.5 . To prepare 500 mL of [M] 0.01 Molarity (M) Tris HCl pH 8.5 , dissolve 605.68 mg Tris in 400 mL deionized water, adjust the pH to pH 8.5 with HCl, and then add water to a final volume of 500 mL [5-8]. Under the hood, syringe filter the PDA solution using a 0.45 μm filter. Treat

the silicone gels with [M] 0.01 % (v/v) PDA solution overnight in the dark at Room temperature .

11 The following day, wash the gels twice with sterile 1X PBS in a sterile hood and sterilize under UV light for 00:30:00 . 30m

Procedure for culturing cells on the gels

12 Make [M] 5 ug/cm^2 Collagen I, Rat Tail **Corning Catalog #**354236 solution by dissolving in sterile 1X PBS. Coat the gels with collagen solution and leave at Room temperature for at least 02:00:00 . After incubation, wash off the collagen with sterile 1X PBS and sterilize under UV light for 00:30:00 . Wells can be stored in sterile 1X PBS at 4 °C until needed [9,10]. 2h 30m

13 Perform trypsinization to detach the cells from the culture vessel. Deactivate the trypsin by adding an appropriate volume of culture media containing serum. Centrifuge the cell suspension to form a cell pellet. Carefully aspirate the supernatant and resuspend the cell pellet in 1 mL of fresh culture media. Count the cells using a hemocytometer or an automated cell counter. Seed the cells into collagen coated wells or petri dishes at the recommended seeding density specific to the cell type being used. Change the culture media as required for the particular cell type to maintain optimal growth conditions.

Microindentation of cells on silicone gels

14 Microindentation is performed using the Chiaro Nanoindenter (Optics 11, the Netherlands). When indenting cells on gels, before each experiment, the probe is calibrated by submerging the cantilever in complete cell media and using a glass calibration dish. The study is performed at Room temperature to prevent measurement error due to temperature drift [4]. The well plates or petri dishes containing the cured gel samples with cells in complete media are mounted onto an Olympus IX73 microscope, thus allowing visualisation of the cells during the experiment under the 10X brightfield setting. Indenting is performed across the wells and petri dishes. The probes used to perform microindentation of cells on gels are within the 0.25 N/m cantilever stiffness and 3 μm radius spherical tip range of probes. An automated 10x6 matrix scan, spaced in 100 μm increments to avoid overlapping, is performed. Ultimately, this process allows for the suitable microindentation of cells.

Fixation and Immunostaining of cells on silicone gels 4h 14m

15 The following steps were applied to human primary astrocytes and leptomeningeal cells for fixation and immunostaining for a 24 well plate. Remove the media and gently wash the cells with 1 mL of 1X PBS containing [M] 0.02 % (v/v) sodium azide. Cover the cells with 20m

500 µL of [M] 4 % (v/v) formaldehyde to fix the cells. Incubate for 00:15:00 at Room temperature. Wash the cells three times with 1 mL 1X PBS containing [M] 0.02 % (v/v) sodium azide for 00:05:00 each. Cells can be stored at 4 °C in 1X PBS containing [M] 0.02 % (v/v) sodium azide until ready for immunostaining.

16 To prepare the cells for immunofluorescent imaging, they were first permeabilized by covering with 500 µL of [M] 0.1 % (v/v) Triton X-100 and incubating at Room temperature for 00:05:00. Subsequently, the cells were washed three times with 1 mL of 1X PBS containing [M] 0.02 % (v/v) sodium azide for 00:05:00 each. Following the washes, the cells were covered with 500 µL of Blocking buffer and incubated at Room temperature for 01:00:00. Remove the blocking solution and add 200 µL of the primary antibody solution to each well. Incubate the cells Overnight at 4 °C. After aspirating the primary antibody solution, the cells were washed three times with 1 mL of 1X PBS containing [M] 0.02 % (v/v) sodium azide for 00:05:00 each. Next, 200 µL of secondary antibody solution was added, and the cells were incubated at Room temperature for 01:00:00. This was followed by aspirating the secondary antibody solution and washing the cells three times with 1 mL of 1X PBS containing [M] 0.02 % (v/v) sodium azide for 00:05:00 each. The cells were then covered with 300 µL of phalloidin solution Phalloidin **Proteintech Catalog #**PF00003 and incubated for 00:20:00 at Room temperature. After this, the cells were washed three times with 1 mL of 1X PBS containing [M] 0.02 % (v/v) sodium azide for 00:05:00 each. Subsequently, the cells were covered with 500 µL of [M] 10 µg/µL DAPI solution and incubated for 00:30:00 at Room temperature. Following the DAPI staining, the cells were washed three times with 1 mL of 1X PBS containing [M] 0.02 % (v/v) sodium azide for 00:05:00 each. Finally, the cells were covered with 1 mL of 1X PBS containing [M] 0.02 % (v/v) sodium azide and imaged on a confocal microscope. 4h 20m

Protocol references

1. Dow Chemical Company T. SYLGARDTM 184 Silicone Elastomer FEATURES & BENEFITS. 2017 [cited 25 Feb 2025]. Available: www.consumer.dow.com

2. SYLGARDTM 184 Silicone Elastomer Kit | Dow Inc. [cited 25 Feb 2025]. Available: https://www.dow.com/en-us/pdp.sylgard-184-silicone-elastomer-kit.01064291z.html#tech-content

3. Dow. SYLGARDTM527 Silicone Dielectric Gel ® TMTrademark of The Dow Chemical Company (“Dow”) or an affiliated company of Dow SYLGARDTM527 Silicone Dielectric Gel. 2017.

4. Optics. PIUMA NANOINDENTER USER MANUAL 2 VISITING ADRESS. [cited 20 Feb 2025]. Available: www.optics11.com

5. Etezadi F, Tuyet Le MN, Shahsavarani H, Alipour A, Moazzezy N, Samani S, et al. Optimization of a PDMS-Based Cell Culture Substrate for High-Density Human-Induced Pluripotent Stem Cell Adhesion and Long-Term Differentiation into Cardiomyocytes under a Xeno-Free Condition. ACS Biomater Sci Eng. 2022;8: 2040–2052. doi:10.1021/ACSBIOMATERIALS.2C00162/SUPPL_FILE/AB2C00162_SI_007.MP4

6. Sharma D, Jia W, Long F, Pati S, Chen Q, Qyang Y, et al. Polydopamine and collagen coated micro-grated polydimethylsiloxane for human mesenchymal stem cell culture. Bioact Mater. 2019;4: 142–150. doi:10.1016/J.BIOACTMAT.2019.02.002

7. Chuah YJ, Koh YT, Lim K, Menon N V., Wu Y, Kang Y. Simple surface engineering of polydimethylsiloxane with polydopamine for stabilized mesenchymal stem cell adhesion and multipotency. Sci Rep. 2015;5: 18162. doi:10.1038/SREP18162

8. Deng Z, Wang W, Xu X, Nie Y, Liu Y, Gould OEC, et al. Biofunction of Polydopamine Coating in Stem Cell Culture. ACS Appl Mater Interfaces. 2021;13: 10748–10759. doi:10.1021/ACSAMI.0C22565/ASSET/IMAGES/LARGE/AM0C22565_0008.JPEG

9. Gelatin Materials. 2009 [cited 25 Feb 2025]. Available: www.corning.com/lifesciences.

10. 354236 | Corning® Collagen I, Rat Tail, 100 mg | Corning. [cited 25 Feb 2025]. Available: https://ecatalog.corning.com/life-sciences/b2c/US/en/Surfaces/Extracellular-Matrices ECMs/Corning%C2%AE-Collagen/p/354236
