# Supplementary material for: A comprehensive protocol for PDMS fabrication for use in cell culture
Source: PLoS One. 2025 May 12;20(5):e0323283. doi: 10.1371/journal.pone.0323283 (PMC12068733; doi:10.1371/journal.pone.0323283)
Supplement: S2 File — Printable version for lab use. (DOCX) [file pone.0323283.s002.docx]

# **A Comprehensive Protocol for PDMS Fabrication for use in Cell Culture**

## **Equipment Setup: Silicone gels**

Gather all required materials and equipment, including silicone base (Part A) and curing agent (Part B) (SYLGARD™ 184 Silicone Elastomer Kit Dow Corning Catalog #04019862 and SYLGARD™ 527 Silicone Dielectric Gel Kit Dow Corning Catalog #2270030 ), weighing scales, stirring rods, Pasteur pipettes, centrifuge tubes, tube rack, weigh boats, syringes, 15mm puncher, well plates, and 60 mm dishes. Set up the equipment under a fume hood or hanging fume hood.


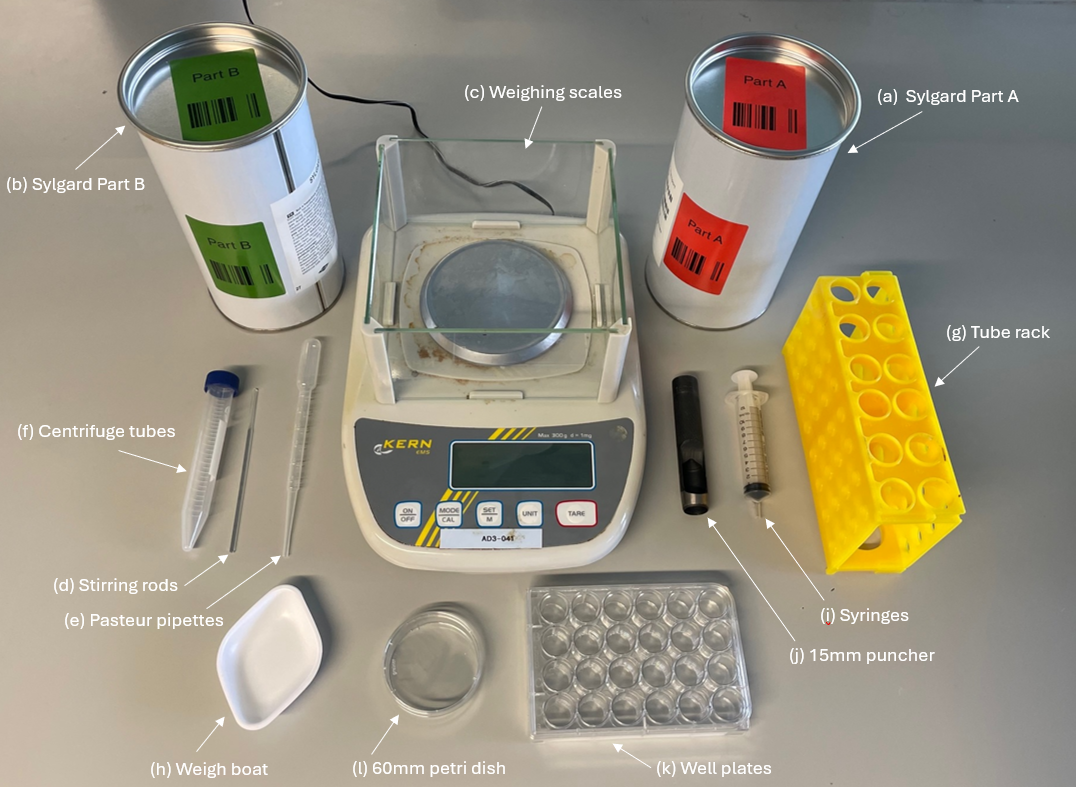


## **Preparation of Silicone Gels**

1. Weigh the desired amounts of base (Part A) and curing agent (Part B) in a weigh boat, ensuring precise measurements for each part.
2. Mix Sylgard 184 and Sylgard 527 using the following mixing procedures.

- For Sylgard 184, stir Part A and Part B for approximately 6 minutes, for masses less than 10 g, with a stirring rod in the weigh boat until thoroughly mixed [1,2]. Increase mixing time as necessary for larger masses. Pour the mixture into a centrifuge tube.
- For Sylgard 527, stir Part A and Part B for 6 minutes, for masses less than 10 g, with a stirring rod in the weigh boat [3]. Transfer the mixture to a centrifuge tube, ensuring centrifuge tubes are filled only halfway to prevent overflow during mixing. Mix vigorously with a pasteur pipette for an additional 8 minutes. Increase mixing times as necessary for larger masses.

1. Centrifuge the silicone mixtures at 600 rcf at 21°C for 5 minutes to remove air bubbles.
2. Cast the Sylgard mixtures into dishes or well plates, ensuring the same mass per well. Refer to Table 1 for recommended volumes for different well plates and petri dishes.

Table 1: Recommended masses for silicone mixtures in various petri dishes and well plates to maintain consistent thickness.

| **Plates / Dishes** | **Surface area (cm²)** | **Silicone mixture (g)** |
| --- | --- | --- |
| 35mm | 8.8 | 1.228 |
| 60mm | 21.5 | 3 |
| 100mm | 56.7 | 7.912 |
| 150mm | 145 | 20.233 |
| 6-well | 9.5 | 1.34 |
| 12-well | 3.83 | 0.53 |
| 24-well | 1.93 | 0.265 |

If air bubbles form during casting, plates can be centrifuged again at 300 rcf at 21°C for 5 minutes.

1. Transfer the silicone gels to an oven and cure at 60 °C for 24-48 hours. Sylgard 184 gels cure within 24 hours, however, some Sylgard 527 gels need 24 hours to cure.

- To increase the stiffness of Sylgard 184 gels, remove the gels from the petri dishes and post-cure at 200 °C for 1 hour (S3 Fig).

## **Microindentation of Silicone gels**

1. Microindentation is performed using the Chiaro Nanoindenter (Optics 11, the Netherlands). As Sylgard 527 gels are adhesive after curing, coat the gels in 5 % (v/v) BSA solution for 2 hours before indenting to prevent the probe from adhering to the gel. Sylgard 184 gels do not require coating with 5 % (v/v) BSA. Before each experiment, calibrate the probe by submerging the cantilever in deionised water and using a glass calibration dish. Conduct the study at room temperature to prevent measurement errors due to temperature drift. Add deionised water to the gels via pipetting to prevent static forces during the indentation process [4]. Perform indentations across the wells and petri dishes. The probes used to perform microindentation of the Sylgard 184 gels are within the 180 N/m cantilever stiffness and 50 µm radius spherical tip range of probes. The probes used to perform microindentation of the Sylgard 527 gels are within the 0.5 N/m cantilever stiffness and 50 µm radius spherical tip range of probes. Execute an automated 5x6 matrix scan, spaced in 1000 μm increments to avoid overlapping. Use the indentation mode with 8000 nm set as the indentation depth and 5000 nm/s as the indentation speed. This process ensures suitable microindentation of silicone gels ranging from 1 kPa to 10 MPa.
2. To perform gel thickness analysis, begin by calibrating the probe with a cantilever stiffness of 180 N/m and a spherical tip with a 50 µm radius. Set the indentation depth to 1 nm. Conduct an automated 6x6 matrix scan, with 1000 µm spacing increments, across an identical empty well plate to the one used for curing the gels. Next, perform the same matrix scan across the Sylgard 184 gels, increasing the indentation depth to 8000 nm. Ensure that the height of the indenter arm remains constant throughout this process. Subsequently, calibrate the probe with a cantilever stiffness of 0.5 N/m, maintaining the spherical tip radius at 50 µm. Execute a matrix scan, with indentation depth set to 1 nm, on the empty well plate once more. Finally, repeat the matrix scan across the Sylgard 527 gels, again increasing the indentation depth to 8000 nm.

## **Procedure for Sterilizing the Silicone Gels after Curing**

For gels cured in well plates or petri dishes, spray the gels with 70% ethanol and then transfer them to a sterile hood. Then add a small amount of 70% ethanol to each well or dish which will be seeded with cells. Without removing the ethanol, sterilize the well plates and petri dishes under UV light in the sterile hood for 30 minutes. After sterilization, remove the ethanol and wash each well and petri dish twice with sterile water.

- If using post-cured gels in 24-well plates, punch out discs from the post-cured gel using a 15mm puncher. Bathe these gel discs in a small amount of 70% ethanol, transfer them to a sterile hood, and wash twice in a sterile water bath. Allow the discs to dry, then insert them into the bottom of the 24-well plates. Add 200 µL of 70% ethanol to each well, sterilize for 30 minutes under UV light in a sterile hood, remove the ethanol, and wash twice with sterile water.

## **Procedure for rendering the gels hydrophilic**

1. Make a 0.01 % (v/v) PDA solution by dissolving 1 mg dopamine hydrochloride in 10 ml of 0.01 Molarity (M) Tris HCl 8.5 . To prepare 500 ml of 0.01 Molarity (M) Tris HCl 8.5 , dissolve 605.68 mg Tris in 400 ml deionized water, adjust the pH to 8.5 with HCl, and then add water to a final volume of 500 ml [5–8]. Under the hood, syringe filter the PDA solution using a 0.45 µm filter. Treat the silicone gels with 0.01 % (v/v) PDA solution overnight in the dark at room temperature.
2. The following day, wash the gels twice with sterile 1X PBS in a sterile hood and sterilize under UV light for 30 minutes.

## **Procedure for culturing cells on the gels**

1. Make 5 ug/cm^2 Collagen I, Rat Tail Corning Catalog #354236 solution by dissolving in sterile 1X PBS. Coat the gels with collagen solution and leave at room temperature for at least 2 hours. After incubation, wash off the collagen with sterile 1X PBS and sterilize under UV light for 30 minutes. Wells can be stored in sterile 1X PBS at 4 °C until needed [9,10].
2. Perform trypsinization to detach the cells from the culture vessel. Deactivate the trypsin by adding an appropriate volume of culture media containing serum. Centrifuge the cell suspension to form a cell pellet. Carefully aspirate the supernatant and resuspend the cell pellet in 1 ml of fresh culture media. Count the cells using a hemocytometer or an automated cell counter. Seed the cells into collagen coated wells or petri dishes at the recommended seeding density specific to the cell type being used. Change the culture media as required for the particular cell type to maintain optimal growth conditions.

## **Microindentation of cells on silicone gels**

Microindentation is performed using the Chiaro Nanoindenter (Optics 11, the Netherlands). When indenting cells on gels, before each experiment, the probe is calibrated by submerging the cantilever in complete cell media and using a glass calibration dish. The study is performed at room temperature to prevent measurement error due to temperature drift [4]. The well plates or petri dishes containing the cured gel samples with cells in complete media are mounted onto an Olympus IX73 microscope, thus allowing visualisation of the cells during the experiment under the 10X brightfield setting. Indenting is performed across the wells and petri dishes. The probes used to perform microindentation of cells on gels are within the 0.25 N/m cantilever stiffness and 3 µm radius spherical tip range of probes. An automated 10x6 matrix scan, spaced in 100 μm increments to avoid overlapping, is performed*.* Ultimately, this process allows for the suitable microindentation of cells.

## **Fixation and Immunostaining of cells on silicone gels**

1. The following steps were applied to human primary astrocytes and leptomeningeal cells for fixation and immunostaining for a 24 well plate. Remove the media and gently wash the cells with 1 ml of 1X PBS containing 0.02 % (v/v) sodium azide. Cover the cells with 500 µL of 4 % (v/v) formaldehyde to fix the cells. Incubate for 15 minutes at room temperature. Wash the cells three times with 1 ml 1X PBS containing 0.02 % (v/v) sodium azide for 5 minutes each. Cells can be stored at 4 °C in 1X PBS containing 0.02 % (v/v) sodium azide until ready for immunostaining.
2. To prepare the cells for immunofluorescent imaging, they were first permeabilized by covering with 500 µL of 0.1 % (v/v) Triton X-100 and incubating at room temperature for 5 minutes. Subsequently, the cells were washed three times with 1 ml of 1X PBS containing 0.02 % (v/v) sodium azide for 5 minutes each. Following the washes, the cells were covered with 500 µL of blocking buffer and incubated at room temperature for 1 hour. Remove the blocking solution and add 200 µL of the primary antibody solution to each well. Incubate the cells overnight at 4 °C. After aspirating the primary antibody solution, the cells were washed three times with 1 ml of 1X PBS containing 0.02 % (v/v) sodium azide for 5 minutes each. Next, 200 µL of secondary antibody solution was added, and the cells were incubated at room temperature for 1 hour. This was followed by aspirating the secondary antibody solution and washing the cells three times with 1 ml of 1X PBS containing 0.02 % (v/v) sodium azide for 5 minutes each. The cells were then covered with 300 µL of phalloidin solution (Phalloidin Proteintech Catalog #PF00003) and incubated for 20 minutes at room temperature . After this, the cells were washed three times with 1 ml of 1X PBS containing 0.02 % (v/v) sodium azide for 5 minutes each. Subsequently, the cells were covered with 500 µL of 10 µg/µL DAPI solution and incubated for 30 minutes at room temperature . Following the DAPI staining, the cells were washed three times with 1 ml of 1X PBS containing 0.02 % (v/v) sodium azide for 5 minutes each. Finally, the cells were covered with 1 ml of 1X PBS containing 0.02 % (v/v) sodium azide and imaged on a confocal microscope.

## **Equipment/materials**

Oven set to 60 °C

Dow Corning Sylgard 184 Part A/B

Dow Corning Sylgard 527 Part A/B

Weighing scales

Plastic stirring rods

Pasteur pipettes

Tube rack

15ml/50ml centrifuge tubes

Weigh boats

10ml syringes

Well plates

60 mm petri dishes

15 mm tissue punch

Centrifuge for tubes and plates

70% ethanol

1X sterile PBS

Laminar flow hood with UV light

Pipetting aid

Serological pipettes

Waste beaker

Dopamine hydrochloride

10 mM Tris-HCl pH 8.5 solution

60 ml syringe

0.45 um syringe filter

Rat tail collagen type 1

## **References**

1. Dow Chemical Company T. SYLGARDTM 184 Silicone Elastomer FEATURES & BENEFITS. 2017 [cited 25 Feb 2025]. Available: www.consumer.dow.com

2. SYLGARDTM 184 Silicone Elastomer Kit | Dow Inc. [cited 25 Feb 2025]. Available: https://www.dow.com/en-us/pdp.sylgard-184-silicone-elastomer-kit.01064291z.html#tech-content

3. Dow. SYLGARDTM527 Silicone Dielectric Gel ® TMTrademark of The Dow Chemical Company (“Dow”) or an affiliated company of Dow SYLGARDTM527 Silicone Dielectric Gel. 2017.

4. Optics. PIUMA NANOINDENTER USER MANUAL 2 VISITING ADRESS. [cited 20 Feb 2025]. Available: www.optics11.com

5. Etezadi F, Tuyet Le MN, Shahsavarani H, Alipour A, Moazzezy N, Samani S, et al. Optimization of a PDMS-Based Cell Culture Substrate for High-Density Human-Induced Pluripotent Stem Cell Adhesion and Long-Term Differentiation into Cardiomyocytes under a Xeno-Free Condition. ACS Biomater Sci Eng. 2022;8: 2040–2052. doi:10.1021/ACSBIOMATERIALS.2C00162/SUPPL_FILE/AB2C00162_SI_007.MP4

6. Sharma D, Jia W, Long F, Pati S, Chen Q, Qyang Y, et al. Polydopamine and collagen coated micro-grated polydimethylsiloxane for human mesenchymal stem cell culture. Bioact Mater. 2019;4: 142–150. doi:10.1016/J.BIOACTMAT.2019.02.002

7. Chuah YJ, Koh YT, Lim K, Menon N V., Wu Y, Kang Y. Simple surface engineering of polydimethylsiloxane with polydopamine for stabilized mesenchymal stem cell adhesion and multipotency. Sci Rep. 2015;5: 18162. doi:10.1038/SREP18162

8. Deng Z, Wang W, Xu X, Nie Y, Liu Y, Gould OEC, et al. Biofunction of Polydopamine Coating in Stem Cell Culture. ACS Appl Mater Interfaces. 2021;13: 10748–10759. doi:10.1021/ACSAMI.0C22565/ASSET/IMAGES/LARGE/AM0C22565_0008.JPEG

9. Gelatin Materials. 2009 [cited 25 Feb 2025]. Available: www.corning.com/lifesciences.

10. 354236 | Corning® Collagen I, Rat Tail, 100 mg | Corning. [cited 25 Feb 2025]. Available: https://ecatalog.corning.com/life-sciences/b2c/US/en/Surfaces/Extracellular-Matrices-ECMs/Corning%C2%AE-Collagen/p/354236
