# Supplementary figures and images for: A comprehensive protocol for PDMS fabrication for use in cell culture
Source: PLoS One. 2025 May 12;20(5):e0323283. doi: 10.1371/journal.pone.0323283 (PMC12068733; doi:10.1371/journal.pone.0323283)

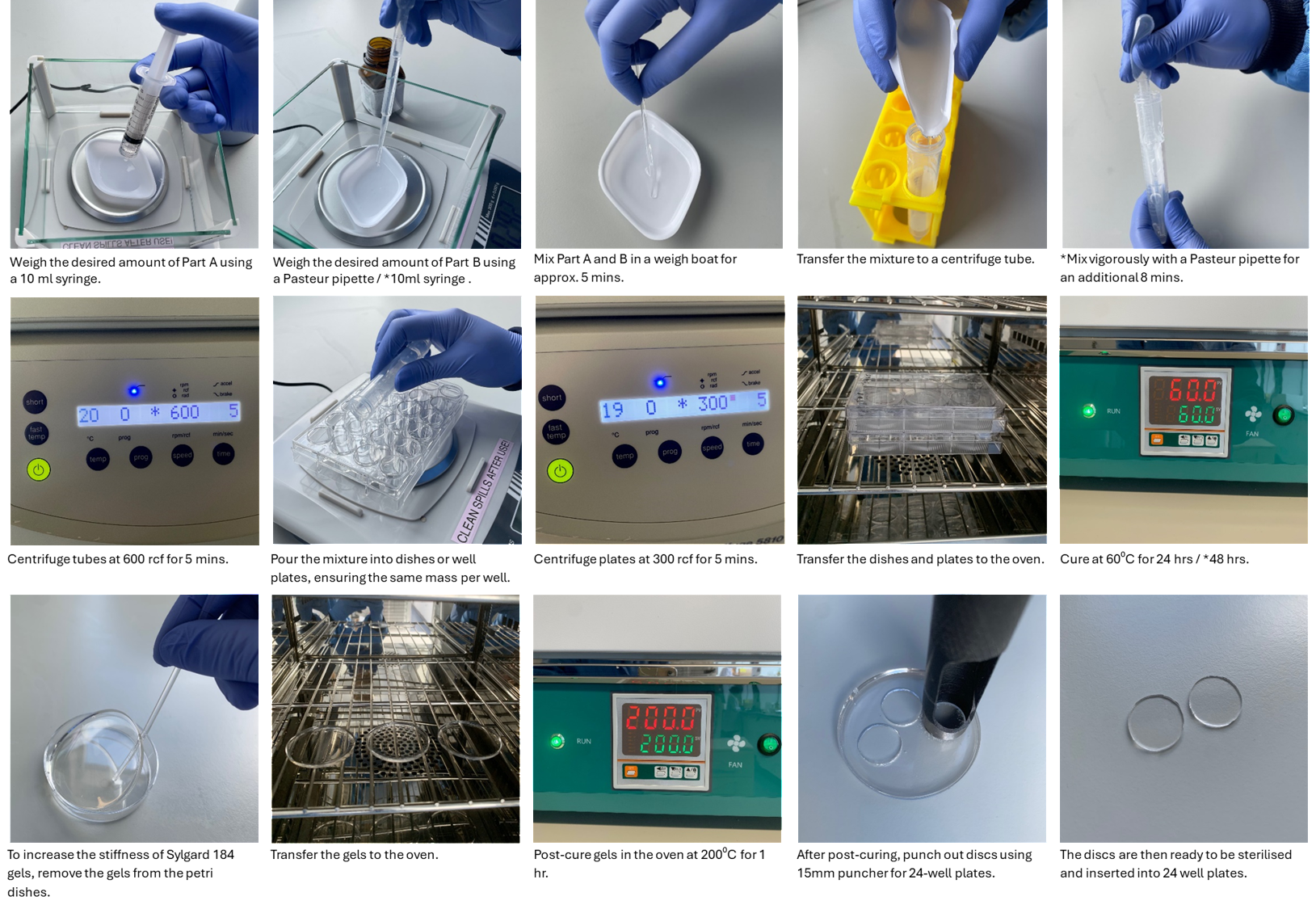

Supplement: S1 Fig — (TIF) [file pone.0323283.s003.tif]

Plastic

|  | S1 | S2 | S3 |
| --- | --- | --- | --- |
| Day 1 | 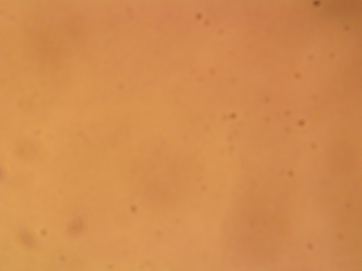 | 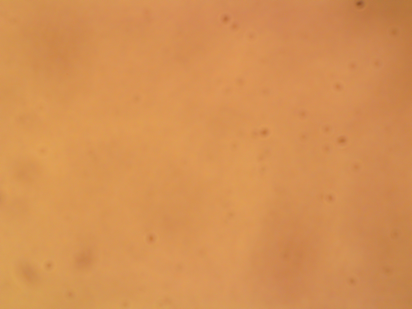 | 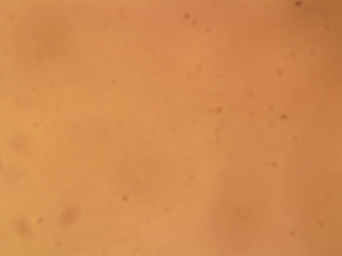 |
| Day 5 | 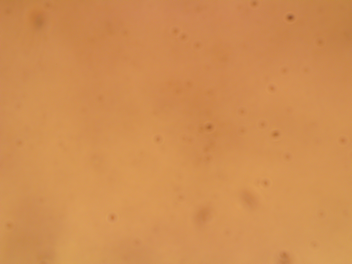 | 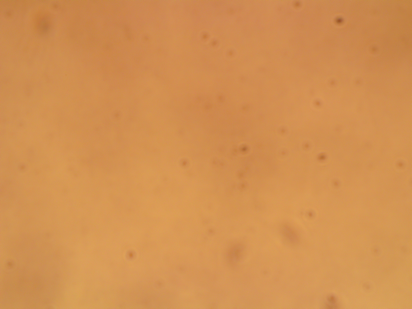 | 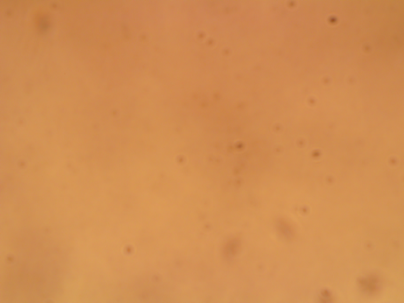 |

Sylgard 184

|  | S1 | S2 | S3 |
| --- | --- | --- | --- |
| Day 1 | 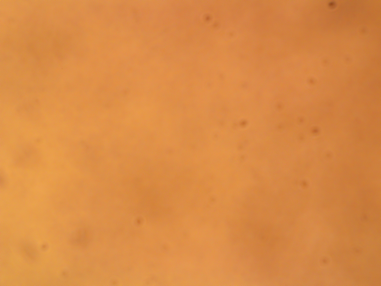 | 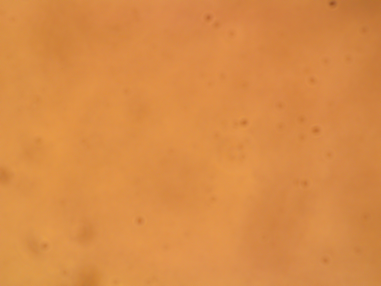 | 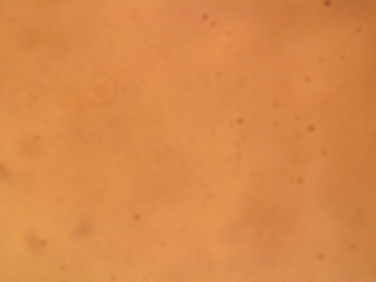 |
| Day 5 | 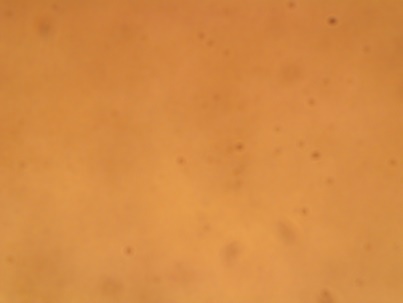 | 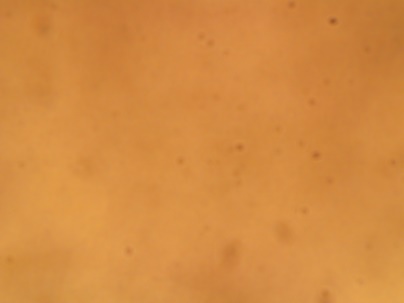 | 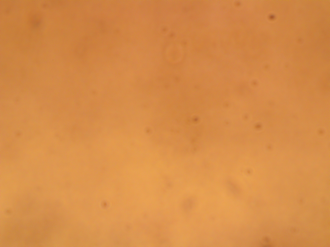 |

Sylgard 527

|  | S1 | S2 | S3 |
| --- | --- | --- | --- |
| Day 1 | 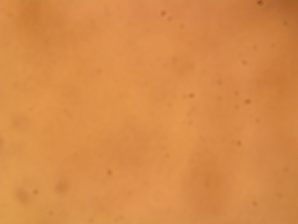 | 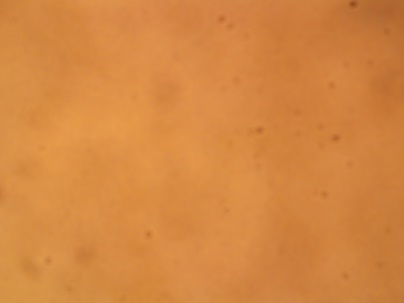 | 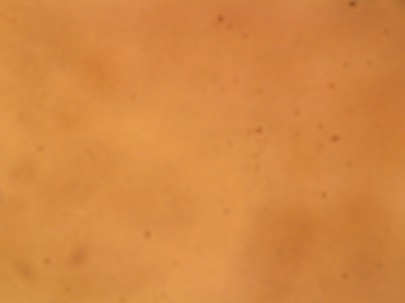 |
| Day 5 | 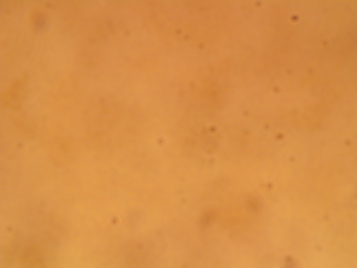 | 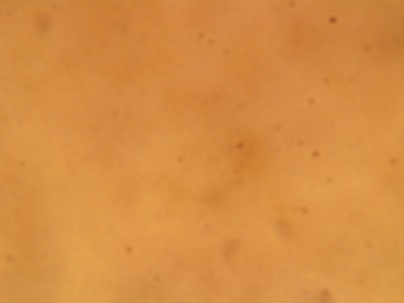 | 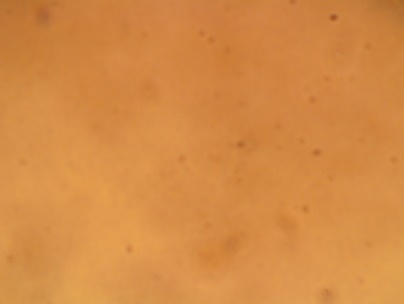 |

Supplement: S3 File — S1, S2, and S3 correspond to images from three separate wells, representing samples one, two, and three, respectively. (DOCX) [file pone.0323283.s006.docx]

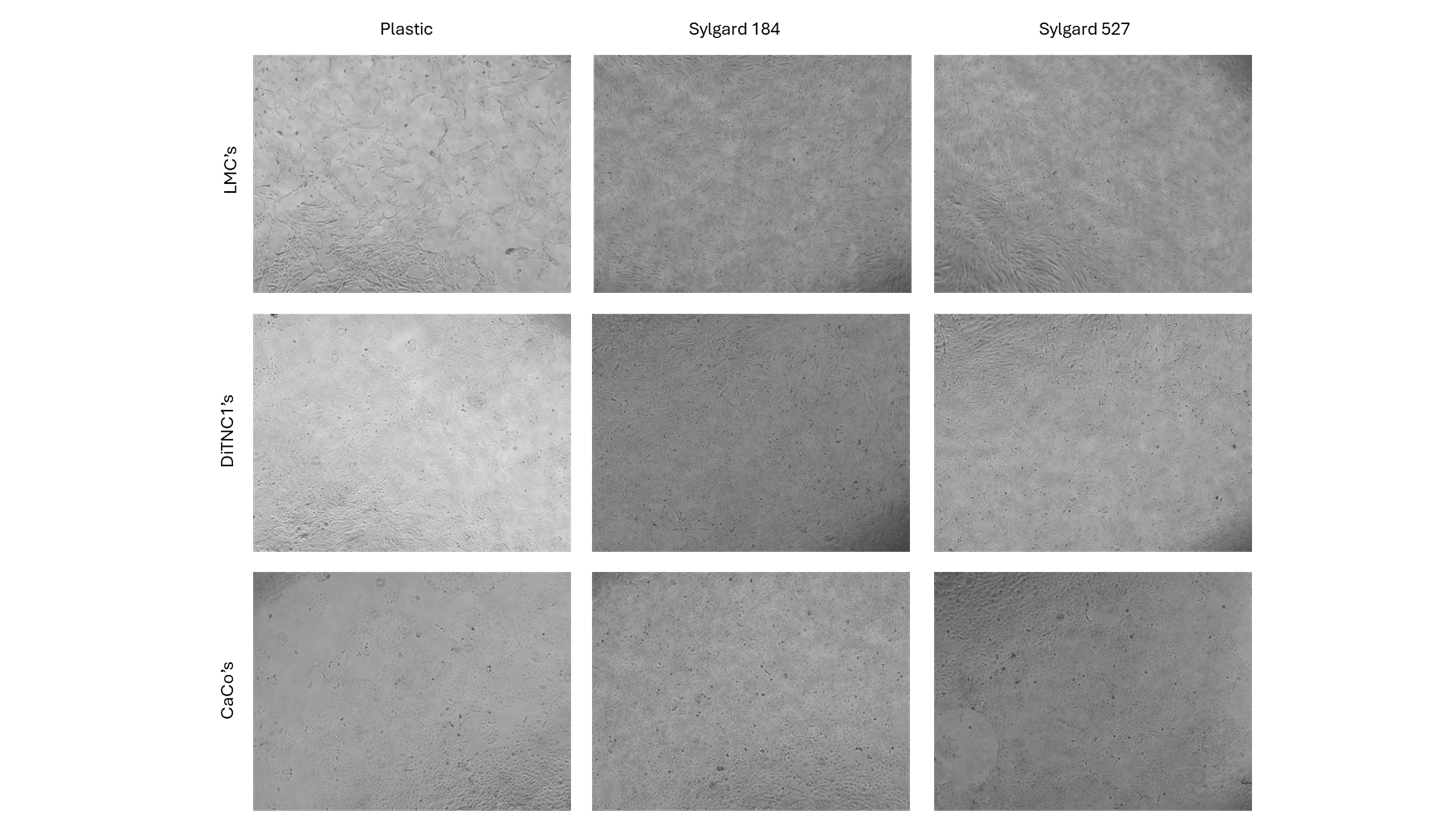

Supplement: S2 Fig — (TIF) [file pone.0323283.s013.tif]

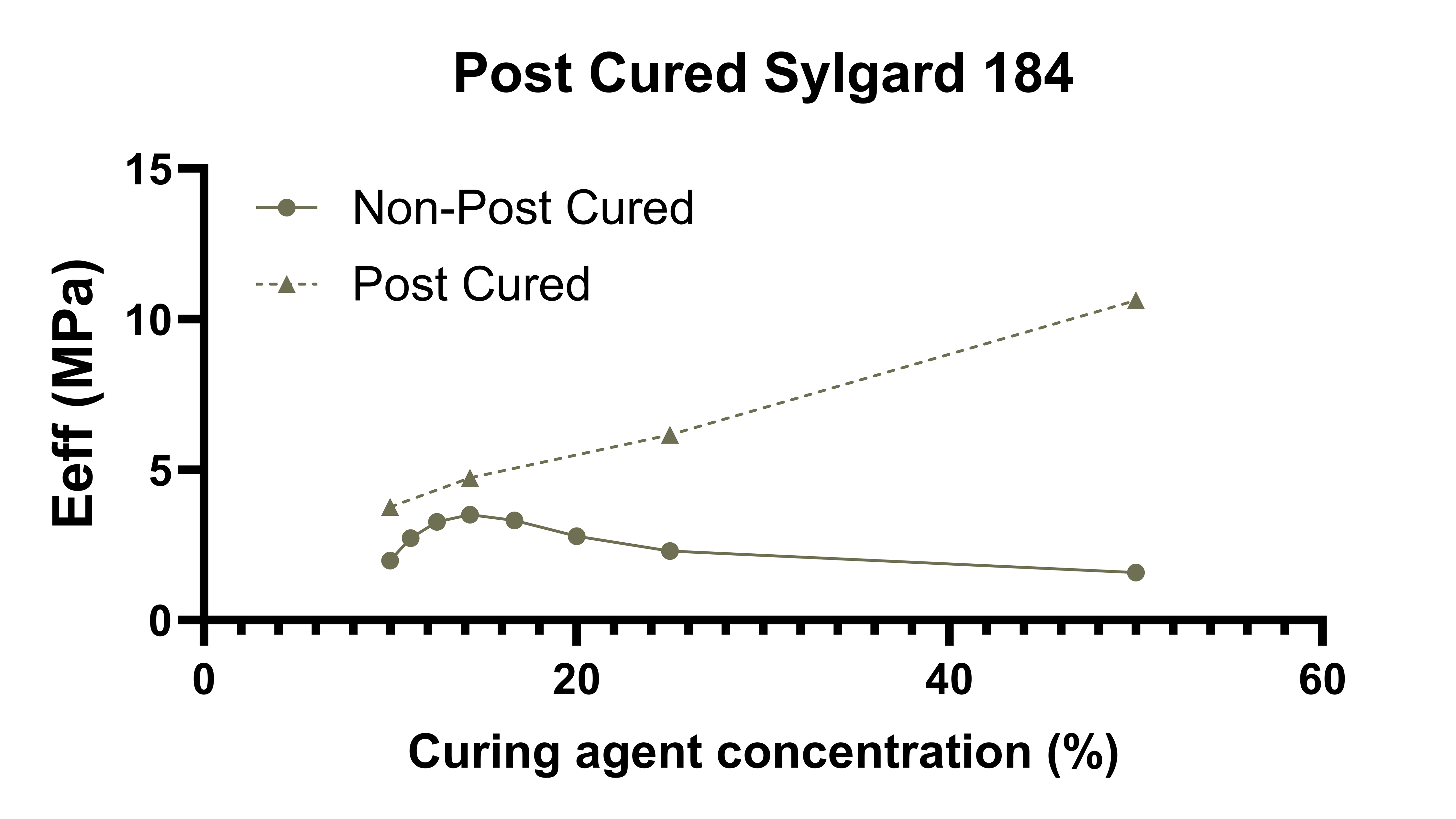

Supplement: S3 Fig — Graph of Sylgard 184 cured at 60⁰C (non-post cured) compared to post cured. After curing at 60°C for 24 hours, Sylgard 184 underwent a post-curing process at 200°C for 1 hour. The results indicate that stiffness increased with higher curing agent concentrations. In the corresponding data, the x-axis represents the curing agent concentration, expressed as the percentage of curing agent (Part B) relative to the base (Part A), while the y-axis denotes the effective elastic modulus. (TIF) [file pone.0323283.s014.tif]
