# Supplementary material for: A comprehensive protocol for PDMS fabrication for use in cell culture
Source: PLoS One. 2025 May 12;20(5):e0323283. doi: 10.1371/journal.pone.0323283 (PMC12068733; doi:10.1371/journal.pone.0323283)
Supplement: S4 File — (DOCX) [file pone.0323283.s007.docx]

**B**

**A**

(A) E*_eff_* values for various masses of Sylgard 184 at a 10:1 ratio. Little to no significant differences were observed between 0.89 and 2.23 grams of gel, indicating that any gel mass within this range is acceptable for consistent stiffness. (B) E*_eff_* values for various masses of Sylgard 527 at a 1:1 ratio. No significant differences were observed between 0.45 and 2.68 grams of gel, suggesting that any gel mass within this range ensures consistent stiffness. Given that the data were not normally distributed, the non-parametric Kruskal-Wallis ANOVA test was used to compare E_eff_ across samples.

| **Mass (g)** | **Sylgard 184 thickness (mm)** | **Sylgard 527 thickness (mm)** |
| --- | --- | --- |
| 0.45 | 0.462 | 0.491 |
| 0.89 | 0.925 | 0.982 |
| 1.34 | 1.387 | 1.472 |
| 1.78 | 1.850 | 1.964 |
| 2.23 | 2.312 | 2.455 |
| 2.68 | 2.774 | 2.946 |
