# Supplementary material for: A comprehensive protocol for PDMS fabrication for use in cell culture
Source: PLoS One. 2025 May 12;20(5):e0323283. doi: 10.1371/journal.pone.0323283 (PMC12068733; doi:10.1371/journal.pone.0323283)
Supplement: Attachment — Submitted filename: Response to Reviewers.docx [file pone.0323283.s016.docx]

The authors wish to thank the reviewers for their input into this manuscript. We believe that the paper has benefited greatly from their insight, helping us to identify the points that needed further clarification for us to enhance the focus and impact of the study.

We have addressed the reviewers’ comments in the following text. All reviewer comments are in **bold**, our responses follow. Additions to the manuscript in response to the reviewers’ comments are in *italics***.**

**Reviewer #1:**

**The authors report a lab protocol to standardize PDMS substrates with tunable stifness for mechaobiology studies for primary cells and cells lines. They discuss operational steps in terms of biocompatibility, mechanical stability,sterilization, autofluorescence and contact angle.
Some points to address:**

1. **Introduce other ways to degas PDMS. In the protocol it is not clear PART A and PART B. Crosslinking agent is missing. Discuss differences between of the two Sylgard gels for applications.**
   - I have made the following inclusions in the Discussion section that addresses the other methods of degassing that can be used in PDMS fabrication. We agree that there are other applicable methods of degassing that can be used.
   - Discussion, page 18, line 423:

*“Following thorough mixing, degassing PDMS is crucial for removing trapped air bubbles and ensuring uniform material properties. The most common approach involves vacuum degassing, either using a vacuum chamber or an oven equipped with a vacuum pump [1–6]. However, alternative methods are available for laboratories without access to such equipment. Centrifugation can facilitate bubble removal by driving air pockets to the surface [7–9]. In the absence of both a vacuum chamber and a centrifuge, allowing the mixture to rest after mixing can promote bubble dissipation.* *In addition to degassing, accurate weighing of the PDMS mixture before curing is essential, as the elastic modulus of PDMS membranes has been shown to decrease with increasing thickness."*

- I have changed the terms PART A and PART B in the protocols.io site in addition to what is mentioned in the manuscript.
- Protocols.io, Step 1:

*“silicone base (Part A) and curing agent (Part B)”*

- Protocols.io, Step 2:

*“amounts of base (Part A) and curing agent (Part B)”*

- Introduction, page 4, line 96:

*“Many studies rely on specialized mixers for combining the silicone base (Part A) and the curing agent (Part B) of PDMS-based elastomers”*

- Discussion, page 18, line 421:

*“For example, mixing the silicone base (Part A) with the curing agent (Part B) without a specialized mixer”*

- The Sylgards were chosen as they have similar chemistry with the main difference being the polymer chain lengths. The difference in chain length is the basis for the differences in mechanical strength (i.e. kPa vs MPa). Furthermore, Sylgard is a common and relatively cheap elastomer that any lab can get access to, which is one of the main objectives of this protocol.
- Introduction, page 3, line 58:

*“Notably, PDMS-based elastomers, such as Sylgard 184 and Sylgard 527, are widely used in biological and engineering research due to their tuneable stiffness, ranging from the kilopascal (kPa) to the megapascal (MPa) scale, which allow them to be used as a mimetic of various biological tissue types. These differences arise from variations in polymer chain lengths and crosslinking densities, allowing researchers to modulate substrate mechanics while maintaining consistent surface chemistry [10]. This flexibility supports diverse applications; Sylgard 184, with a modulus in the MPa range, is suited for culturing cells that reside in rigid environments, such as fibroblasts, endothelial cells, and osteoblasts [11–14]. It is also widely used in microfluidics, soft lithography, and electronic encapsulation due to its durability and chemical resistance* *[15–17]. Sylgard 527, with a kPa-range modulus, is ideal for softer substrates used in stem cell and myoblast cultures [18–20] and is suitable for sealing and protecting various electronic devices, especially those with delicate components [21].”*

2. **What is contact angle if you activate by oxygen plasma substrates before PDA? Why do you choose only PDA and concentration?**
   - We do not have an oxygen plasma treater in our lab so looking at the contact angle after oxygen plasma treatment was not possible. Furthermore, we would stay using PDA as a technique as it aligns with the study objective of using widely available lab tools to carry out this protocol. However, we have attempted to address the comment by analysing other PDA concentrations that are commonly used for cell culture (i.e. 0.1 – 2 mg/ml).

- We tested different PDA concentrations on Sylgard 184 10:1 gels and found that there were no significant differences in contact angle between the three PDA concentrations and so choose to use 0.01% PDA for testing all cell types included in the paper in order to ensure the gels had good transparency for downstream immunofluorescent staining. All cell lines tested in this study adhered well using the 0.01% PDA concentration as well as 5ug/ml collagen coating seen from the data in Fig 10. Of course, the PDA concentrations would need to be optimised for one’s own cells to ensure cell adhesion. All PDA concentrations were left on the substrates at room temp for 24hrs.

- Results, page 13, line 298:

*“Given that the data were normally distributed, the Welch’s t test was used to compare the contact angles of water droplets on uncoated PDMS versus 0.01% PDA-coated PDMS.”*

- Discussion, page 20, line 462:

*“In this study, PDA was selected as the surface coating to render the PDMS substrates hydrophilic due to its easy application. In cell culture applications, PDA coatings are typically prepared using dopamine concentrations between 0.1 mg/ml and 2 mg/ml. Lower concentrations, such as 0.1 mg/ml (0.01% w/v), have been shown to enhance cell adhesion, proliferation, and differentiation on PDMS substrates, with coating durations ranging from 1 hour to overnight, followed by rinsing and sterilization [22–25]. Similarly, certain cell types can proliferate effectively on higher PDA concentrations (>0.01% w/v), where thicker coatings enhance surface functionalization [26–29]. Thus, optimizing PDA concentration is essential for achieving the desired cellular responses. This study confirms that PDA enhances the hydrophilicity of PDMS substrates, as evidenced by reduced water contact angles, which is crucial for promoting robust cell adhesion.”*

3. **How do you ensure flatness and uniformity of films in the same well? Explain better why there are statistical differences between samples.**
   - The flatness and uniformity of the PDMS samples were assessed using topographical data obtained from nanoindentation. Since the topographical data from nanoindentation shows a consistent tilt in both the plastic dish and the PDMS samples, we can conclude that this tilt is due to the positioning of the well plate on the nanoindenter stage rather than an inherent unevenness in the samples. By accounting for the tilt observed in the plastic dish (comparing to the plastic control where no gels are applied), it becomes evident that the PDMS samples themselves are flat and uniform. We attempted to remove the effects of the tilt within the system but at that scale it is difficult to remove these slight tilt effects (i.e. 1-2˚can cause the images you see below).

|  | Sylgard 184 10:1 | Sylgard 527 1:1 |
| --- | --- | --- |
| Plastic |  |  |
| Sample 1 |  |  |
| Sample 2 |  |  |
| Sample 3 |  |  |

- Results, page 10, line 240:

*“The z-height data obtained from the nanoindenter revealed statistically significant differences between the samples cast at different times. This statistical significance arises primarily from the combination of a low standard deviation within each group and the relatively large sample size (n = 30 per group), which increases the power of the statistical test to detect even very small differences.*

*While the test detects a difference, the tightly clustered distributions within each sample group suggest that the actual variation in PDMS thickness is minimal. This indicates that the statistical significance is likely an artifact of high test sensitivity rather than a meaningful difference in thickness of the samples. Such effects are common when within-group variability is small, as even slight systematic variations can produce statistically significant results without practical relevance.*

*Importantly, the compact distribution of data points within each sample group confirms that thickness variations across individual samples are minimal, further supporting the flatness and uniformity of the films. This consistency underscores the reliability of the casting process and highlights the suitability of these substrates for cell culture applications, where surface uniformity is essential. Figure 3 presents the thickness measurements, and the raw data can be found in Supporting Document S2 Dataset accompanying this article.”*

- Results, page 11, line 275:

*“Despite the statistical significance observed due to low within-group variability and a large sample size (n = 30 per group), the tightly clustered distributions indicate minimal actual variation in PDMS thickness. This suggests that the detected differences are likely a result of high test sensitivity rather than meaningful deviations in sample thickness.”*

4. **Which is the minimum uniform thickness you are able to obtain with casting on plates? Is it compatible with confocal analysis?**
   - The minimum uniform thickness achievable with casting on plates was determined to be 0.45 g (by mass as this is the most quantifiably consistent method of fabricating gels) of PDMS per well in a 6-well plate, as shown in Figure 4. This mass equates to a thickness of 0.463 mm in Sylgard 184 and 0.491 mm in Sylgard 527. Below this mass, it becomes challenging to achieve full coverage of the well bottom, making it difficult to form a uniform film thickness. The table below provides the theoretical thickness values corresponding to each mass of PDMS used.

| **Mass (g)** | **Sylgard 184 thickness (mm)** | **Sylgard 527 thickness (mm)** |
| --- | --- | --- |
| 0.45 | 0.462 | 0.491 |
| 0.89 | 0.925 | 0.982 |
| 1.34 | 1.387 | 1.472 |
| 1.78 | 1.850 | 1.964 |
| 2.23 | 2.312 | 2.455 |
| 2.68 | 2.774 | 2.946 |

- Furthermore, the topography data for the minimum thickness, although showing a slight slant, aligns with the slant observed in the topography data from the plastic plate. This tilt is consistent and attributed to the positioning of the plate on the stage of the nanoindenter.

| Sylgard 184 0.45g | Plastic |
| --- | --- |
|  |  |

- Regarding compatibility with confocal analysis, any of the obtained thicknesses would be suitable, provided the appropriate settings are used on the confocal microscope. Specifically, a z-stack approach can be employed to accurately locate cells on the PDMS substrate, ensuring that imaging can be performed effectively across different depths of the sample.

5. **Discuss the effect of curing temperature on elastic modulus.**
   - Good point that is important to include when discussing PDMS fabrication. We include a sentence in the introduction about effects of curing temperature with references. Also, in the context of this study we have two criteria necessary when fabricating that dictate our choice of curing temperature; effect of temperature on plate (max 60 ℃) and consistency of curing. Please see the figure below to demonstrate the supplementary work we have carried to determine the effect of curing temperature, as well as post-cure temperature, on Sylgard 184 stiffness. We did not include this work as it was outside the remit of the study.
   - Introduction, page 4, line 84:

*“Sylgard 184 exhibits an increase in stiffness with higher curing temperatures due to enhanced crosslinking, with its elastic modulus ranging from ~1 to ~4 MPa between 25°C and 200°C [30,31]. In contrast, Sylgard 527 remains highly compliant, with negligible changes in elastic modulus, maintaining values within the low kPa range regardless of curing temperature [32,33].”*

- Discussion, page 18, line 414:

*“By carefully controlling the mixing ratios and curing conditions, the study demonstrated that consistent mechanical properties could be achieved across different batches of PDMS substrates. To prevent thermal deformation of the plastic culture dishes, which could not withstand higher temperatures, PDMS samples were cured at 60°C. This curing process also reduced curing time and ensured uniform substrate properties. Maintaining this consistency is crucial, as even minor variations in substrate stiffness can significantly affect cell behaviour, including adhesion, proliferation, and differentiation.”*

- After curing at 60°C for 24 hours, Sylgard 184 underwent a post-curing process at 200°C for 1 hour. The results indicate that stiffness increased with higher curing agent concentrations. In the corresponding data, the x-axis represents the curing agent concentration, expressed as the percentage of curing agent (Part B) relative to the base (Part A), while the y-axis denotes the effective elastic modulus.

**Reviewer #2:**

**The authors have described a protocol for fabricating PDMS with different stiffnesses for examining the effect of stiffness on cells. Some clarifications are required.**

1. **Was mechanical testing performed before or after sterilization and treatment with PDA and collagen? Do these processes have any effect on the mechanical properties of the surface on which the cells adhere?**
   - Great question and one we were curious about. We carried out the following experiment to address this question.
   - Mechanical characterization was conducted prior to sterilization and subsequent treatment with PDA and collagen. The mechanical properties of PDMS samples were assessed under three conditions: uncoated (no PDA or collagen), PDA-coated, and PDA and collagen coated. No statistically significant differences were observed among these conditions for Sylgard 184 at a 7:1 concentration. However, for Sylgard 527 at a 1:0.82 concentration, statistical significance was detected (p < 0.05). Despite this, the observed stiffness variation (~5–7 kPa) falls within the range of batch-to-batch variability associated with differences in preparation time, as shown in Figure 2. Therefore, the statistical significance likely reflects methodological variability rather than a true difference in material properties.

2. **Do the different PDMS formulations undergo relaxation during indentation? Is the material purely elastic or does it have a viscoelastic response?**
   - A valid question that we had already carried out but did not include in the original submission. As part of the indentation testing protocol, we do acquire elastic/viscoelastic response. We see from the figure below for Sylgard 184 that there is no relaxation over the course of the load being applied indicating that the material is not relaxing. However, there is some relaxation in the Sylgard 527 concentrations 1:0.65, 1:0.75, and 1:0.82 but this is expected for such soft substrates when the crosslinking agent is reduced. We argue that even for the lower concentrations of 527 that the drop in load is negligible in the kPa range.

|  | Sylgard 184 |  | Sylgard 527 |
| --- | --- | --- | --- |
| 2:1 |  | 1:0.65 |  |
| 4:1 |  | 1:0.75 |  |
| 5:1 |  | 1:0.82 |  |
| 6:1 | ‘ | 1:0.9 |  |
| 7:1 |  | 1:1 |  |
| 8:1 |  | 1:1.1 |  |
| 9:1 |  | 1:1.25 |  |
| 10:1 |  |  |  |

3. **In the protocol, it is mentioned that collagen is used to coat the surface, but this is not included in the manuscript. Can the authors make it clear if collagen was used before adding cells. In the protocol, it also doesn't state what type of collagen is used or how it was dissolved (e.g. is it in acidic acid?)**
   - Good point that we left this key detail out, we initially left this out because our focus was primarily on rendering the gels hydrophilic, which was achieved using PDA. However, in hindsight, the collagen coating is an important aspect of the protocol. We include the below text in the results section to address this point.
   - Results, page 12, line 281:

*“The improved wettability facilitates the adsorption of biomolecular coatings such as collagen, poly-L-lysine, or fibronectin, which are critical for promoting cell adhesion, proliferation, and function. The strong adhesive properties of PDA enable tailored biochemical functionalization, making it a versatile strategy for optimizing cell-material interactions. Contact angle measurements confirmed that PDA-coated and PDA-collagen-coated substrates exhibited significantly lower contact angles compared to uncoated PDMS, indicating superior hydrophilicity (Fig. 4).”*

- Discussion, page 20, line 471:

*“This improved wettability facilitates the efficient adsorption of biomolecular coatings, further supporting cell adhesion, proliferation, and function, key factors for successful cell culture experiments. The biocompatibility of PDA-collagen-coated PDMS seeded with cells was validated through AlamarBlue assays and live/dead staining, confirming that cell viability and proliferation were comparable to those on standard plastic culture plates. These results underscore the effectiveness of this approach in modifying PDMS surfaces to support cell viability and function."*

- Limitations and Future Directions, page 22, line 529:

*“Additionally, while PDA coating was demonstrated to enhance the hydrophilicity of PDMS, thereby supporting the adsorption of biomolecular coatings, there remains an opportunity to explore alternative surface modification techniques.”*

- I have updated the information on the type of collagen used in the protocols.io.
- Step 13:

*“Make 5 ug/cm^2 Collagen I, Rat Tail Corning Catalog #354236 solution by dissolving in sterile 1X PBS. Coat the gels with collagen solution and leave at Room temperature for at least 02:00:00 .”*

4. **More information or a reference is required for how you examined microbial growth. Did your media contain antibiotics or antimitotics? Were they incubated at 37C? How long was the test performed for?**
   - We have now included a reference on using media as indicator of microbial infection [34,35].
   - We used a basic cell culture technique to test sterility by leaving DMEM, a pH-sensitive medium containing phenol red, on the substrates in the incubator overnight. Phenol red serves as a pH indicator, changing from red (neutral pH) to yellow (acidic pH) in response to waste products from microbial growth, such as bacteria or fungi. Contamination is indicated by acidification, a yellow colour change, and increased turbidity, while sterile conditions are confirmed by the absence of turbidity, microbial colonies, or colour change. This simple yet effective method provides an alternative to antimicrobial testing for ensuring the substrates are contamination-free and suitable for cell culture. Again, the premise of using such a simple method is based on our overall objective of using widely available protocols as not every lab will have a microbial culture facility.
   - Expected results, page 6, line 139:

*“The sterilization protocol must be highly effective in eliminating all forms of microbial contamination, ensuring that the PDMS substrates remain sterile throughout the experimental period. To verify sterility, DMEM containing 10% FBS, 2% L-glute, 1% P/S will be added to the sterilized substrates within the wells and incubated at 37°C for 5 days. The absence of contamination will be confirmed by monitoring for any signs of microbial growth over the 5 day incubation period [35]. This will be evidenced by the lack of turbidity, absence of colour change, and no observable microbial colonies in the media [34].”*

- Results, page 14, 335:

*“Fig 5. Assessment of the sterility of PDA-coated PDMS substrates through colour change analysis over a 5-day incubation period.”*

5. **For contact angle measurements after treatment with PDA, were these values consistent between all the different PDMS substrates? This data should be shown. Similarly, was contact angle examined after collagen coating?**

- Contact angle measurements were conducted on all PDMS substrates, including uncoated PDMS, 0.01% PDA-coated PDMS, and 0.01% PDA/Collagen-coated PDMS. The uncoated PDMS exhibited the highest contact angle, measuring approximately 110 ± 15°, indicating its inherent hydrophobicity. PDA-coated substrates showed a reduced contact angle of approximately 65 ± 10°, demonstrating increased hydrophilicity due to PDA deposition. Further treatment with collagen on PDA-coated PDMS resulted in an even lower contact angle of around 45 ± 15°, suggesting an additional enhancement in surface hydrophilicity. Regarding the consistency of contact angle values across different PDMS substrates following PDA treatment, the measurements remained within a comparable range, though minor variations were observed. Additionally, contact angle measurements were conducted after collagen coating, confirming its further impact on surface wettability. These findings highlight the progressive increase in hydrophilicity following surface modifications, which is crucial for optimizing cell adhesion and culture conditions.

**Reviewer #3:**

**Authors developed a standardized method for fabricating PDMS substrates with tunable stiffness, ranging from kPa to MPa, suitable for diverse cell types using standard laboratory equipment in their manuscript. The protocol will be very useful for the cell culture audience. However, before publication some points need to be clarified.**

1. **Authors should provide electron microscopy images before cell culture studies, surface morphology has an effect on cell behavior and should be proven that same for all samples**.
   - Scanning electron microscopy (SEM) was performed on Sylgard 184 (10:1) and Sylgard 527 (1:1), which are the manufacturer’s recommended formulations, as well as on tissue culture-treated plastic. While all coatings exhibit some imperfections, these are attributed to handling prior to imaging rather than inherent material defects. In particular, Sylgard 527 is an adherent gel by nature and as part of the SEM process, we had to press the gel on the carbon tape, what you see on the 527 is the residue from the glove leaving an imprint on the gel. The scale bar in all images represents 100 µm.

|  | No Coating | PDA+Collagen Coating |
| --- | --- | --- |
| TC treated plastic |  |  |
| Sylgard 184 10:1 |  |  |
| Sylgard 527 1:1 |  |  |

2. **What is the stability of PDA coating please discuss**
   - Good question, we conducted an experiment to address this.
   - To assess the stability of the surface properties, 0.01% PDA was added to samples of Sylgard 184 (10:1) and Sylgard 527 (1:1), following the manufacturer’s recommended concentrations. Contact angle measurements were taken 24 hours after PDA was introduced (Day 1). The samples were then submerged in water and incubated at 37°C, with additional contact angle measurements recorded on Days 4 and 7. As observed in the graph below, there are no significant changes in contact angle over time, indicating that the surface properties of the material remain stable. This suggests that the wettability and hydrophobicity of the surface are well maintained, with minimal degradation or alteration in surface chemistry. The consistency in contact angle measurements further supports the material’s suitability for applications requiring long-term stability and reliable surface interactions.

3. **Please provide data for PDA surface coverage percentages.**
   - PDA was applied to the entire surface of the gels; however, we were unable to directly quantify the percentage of PDA coverage. Indirect evidence of successful PDA coating comes from contact angle measurements, which showed a reduction from ~100° on non-coated PDMS to ~60° on the PDA-coated PDMS. This change in contact angle brought the surface properties of the PDA-coated gels in line with those observed on TC-treated plastic, indicating effective surface modification.
4. **Toxicity and proliferation data might give ideas about cell behavior. However, it does not represent that cells are healthy. Please use different words instead of cell health. More studies are needed to make this comment.**
   - Good point, and we should have been more careful using our terms throughout. We have changed ‘cell health’ to use “cell viability” as this better reflects the data we have obtained. Below are the specific sentences where I have changed these terms.
   - Expected results, page 7, line 154:

*“This assay aims to evaluate the proliferation and viability of cells cultured under various substrate stiffness conditions.”*

- Expected results, page 7, line 157:

*“This confirmation will serve to validate the cellular viability status of cells cultured on sterile PDMS substrates. Key outputs of this criterion encompass verifying sterility through pH colour change confirmation, assessing hydrophilicity via contact angle measurements, and evaluating cellular viability and metabolic activity using AlamarBlue analysis.”*

- Results, page 13, line 312:

*“The sterile environment provided by the PDMS substrates is critical for maintaining cell viability and ensuring the accuracy of experimental results, thereby supporting their use in various cell culture applications.”*

- Results, page 14, line 327:

*“Cell Viability”*

- Result, page 16, line 389:

*“As evidenced above by the Alamar Blue assay and Live/Dead staining results, multiple cell types remained viable on both Sylgard 184 and Sylgard 527 substrates.”*

- Result, page 17, line 396:

*“The findings confirm that all these cell types adhered well and grew robustly on the PDMS substrates, showcasing their reliability and suitability for a wide range of cell culture applications without adversely affecting cell viability.”*

- Discussion, page 20, line 476:

*“These results underscore the effectiveness of the protocol in preparing PDMS surfaces that support cell viability and function.”*

5. **Since this is a protocol for standardization, why did not the authors perform cell studies using a cell type from 10993-5 ISO.**

- Three different cell types were tested in this study: primary human leptomeningeal cells, an immortalized cell line (DiTNC1), and a cancerous cell line (CaCo-2). All three cell types exhibited good proliferation and viability on the PDMS substrates, demonstrating the suitability of the protocol for cell culture applications. The decision not to use a cell type specified in ISO 10993-5 was based on the intended purpose of this protocol. ISO 10993-5 outlines standardized methods for assessing the in vitro cytotoxicity of medical devices, whereas this protocol is designed for cell culture applications. Its primary objective is to establish a standardized method that enables researchers to modify substrate stiffness on plastic culture plates, allowing for more physiologically relevant cell culture conditions. This approach facilitates the study of how various cell types respond to different mechanical environments, rather than serving as a direct cytotoxicity assessment under ISO 10993-5 guidelines. However, if we did have access to the cell types listed in the ISO we would have applied them to the gels, but unfortunately due to the time and financial constraints we were unable to acquire the cell lines to apply to the gel. To address this comment, we added the following statement to the Discussion section.
- Discussion, page 21, line 490:

*"While ISO 10993-5 specifies cell lines for cytotoxicity assessments, this study focused on establishing a protocol for modulating substrate stiffness to create physiologically relevant cell culture conditions. Therefore, primary human leptomeningeal cells, an immortalized cell line (DiTNC1), and a cancerous cell line (CaCo-2) were selected based on their relevance to the study objectives. This broad selection ensures that the method outlined in this study is applicable across different cellular contexts and provides insight into how various cell types adhere to and proliferate on PDMS substrates using this approach.”*

- The next step for utilising this protocol is to apply the cell lines listed in ISO10993-5 to be applied to the gels to determine their viability on these gels.

1.	Morbioli GG, Speller NC, Stockton AM. A practical guide to rapid-prototyping of PDMS-based microfluidic devices: A tutorial. Anal Chim Acta. 2020;1135: 150–174. doi:10.1016/J.ACA.2020.09.013

2.	Kaigala G V., Ho S, Penterman R, Backhouse CJ. Rapid prototyping of microfluidic devices with a wax printer. Lab Chip. 2007;7: 384–387. doi:10.1039/B617764F

3.	Morbioli GG, Speller NC, Cato ME, Cantrell TP, Stockton AM. Rapid and low-cost development of microfluidic devices using wax printing and microwave treatment. Sens Actuators B Chem. 2019;284: 650–656. doi:10.1016/J.SNB.2018.12.053

4.	Bartholomeusz DA, Boutté RW, Andrade JD. Xurography: Rapid prototyping of microstructures using a cutting plotter. Journal of Microelectromechanical Systems. 2005;14: 1364–1374. doi:10.1109/JMEMS.2005.859087

5.	McDonald JC, Chabinyc ML, Metallo SJ, Anderson JR, Stroock AD, Whitesides GM. Prototyping of microfluidic devices in poly(dimethylsiloxane) using solid-object printing. Anal Chem. 2002;74: 1537–1545. doi:10.1021/AC010938Q/ASSET/IMAGES/LARGE/AC010938QF00008.JPEG

6.	Duffy DC, McDonald JC, Schueller OJA, Whitesides GM. Rapid prototyping of microfluidic systems in poly(dimethylsiloxane). Anal Chem. 1998;70: 4974–4984. doi:10.1021/AC980656Z/ASSET/IMAGES/LARGE/AC980656ZF00008.JPEG

7.	LaFratta CN, Baldacchini T, Farrer RA, Fourkas JT, Teich MC, Saleh BEA, et al. Replication of two-photon-polymerized structures with extremely high aspect ratios and large overhangs. Journal of Physical Chemistry B. 2004;108: 11256–11258. doi:10.1021/JP048525R/ASSET/IMAGES/LARGE/JP048525RF00004.JPEG

8.	Mazzeo AD, Lustrino ME, Hardt DE. Bubble removal in centrifugal casting: Combined effects of buoyancy and diffusion. Polym Eng Sci. 2012;52: 80–90. doi:10.1002/PEN.22049

9.	Degas PDMS in two minutes – Chips and Tips. [cited 17 Feb 2025]. Available: https://blogs.rsc.org/chipsandtips/2010/08/17/degas-pdms-in-two-minutes/?doing_wp_cron=1722405547.1149621009826660156250

10.	Palchesko RN, Zhang L, Sun Y, Feinberg AW. Development of Polydimethylsiloxane Substrates with Tunable Elastic Modulus to Study Cell Mechanobiology in Muscle and Nerve. PLoS One. 2012;7: e51499. doi:10.1371/JOURNAL.PONE.0051499

11.	Park SA, Kim IA, Lee YJ, Shin JW, Kim CR, Kim JK, et al. Biological responses of ligament fibroblasts and gene expression profiling on micropatterned silicone substrates subjected to mechanical stimuli. J Biosci Bioeng. 2006;102: 402–412. doi:10.1263/JBB.102.402

12.	Erenay B, Sağlam ASY, Garipcan B, Jandt KD, Odabaş S. Bone surface mimicked PDMS membranes stimulate osteoblasts and calcification of bone matrix. Biomaterials Advances. 2022;142: 213170. doi:10.1016/J.BIOADV.2022.213170

13.	Park JY, Yoo SJ, Lee EJ, Lee DH, Kim JY, Lee SH. Increased poly(dimethylsiloxane) stiffness improves viability and morphology of mouse fibroblast cells. Biochip J. 2010;4: 230–236. doi:10.1007/S13206-010-4311-9/METRICS

14.	Hassanisaber H, Jafari L, Campeau MA, Drevelle O, Lauzon MA, Langelier E, et al. The effect of substrate bulk stiffness on focal and fibrillar adhesion formation in human abdominal aortic endothelial cells. Materials Science and Engineering: C. 2019;98: 572–583. doi:10.1016/J.MSEC.2018.12.130

15.	Johnston ID, McCluskey DK, Tan CKL, Tracey MC. Mechanical characterization of bulk Sylgard 184 for microfluidics and microengineering. Journal of Micromechanics and Microengineering. 2014;24: 035017. doi:10.1088/0960-1317/24/3/035017

16.	Schmid H, Michel B. Siloxane polymers for high-resolution, high-accuracy soft lithography. Macromolecules. 2000;33: 3042–3049. doi:10.1021/MA982034L/ASSET/IMAGES/LARGE/MA982034LF00004.JPEG

17.	Nedoma J, Fajkus M, Martinek R, Novak M, Zboril O, Jargus J, et al. Influence of different encapsulation types and shapes of polydimethylsiloxane on the temperature sensitivity of the FBG. https://doi.org/101117/122256716. 2017;10208: 195–202. doi:10.1117/12.2256716

18.	Iberite F, Salerno M, Canale C, Rosa A, Ricotti L. Influence of substrate stiffness on human induced pluripotent stem cells: Preliminary results. Proceedings of the Annual International Conference of the IEEE Engineering in Medicine and Biology Society, EMBS. 2019; 1039–1043. doi:10.1109/EMBC.2019.8857397

19.	Rathod ML, Ahn J, Saha B, Purwar P, Lee Y, Jeon NL, et al. PDMS Sylgard 527-Based Freely Suspended Ultrathin Membranes Exhibiting Mechanistic Characteristics of Vascular Basement Membranes. ACS Appl Mater Interfaces. 2018;10: 40388–40400. doi:10.1021/ACSAMI.8B12309/ASSET/IMAGES/LARGE/AM-2018-12309M_0010.JPEG

20.	MacNearney D, Mak B, Ongo G, Kennedy TE, Juncker D. Nanocontact Printing of Proteins on Physiologically Soft Substrates to Study Cell Haptotaxis. Langmuir. 2016;32: 13525–13533. doi:10.1021/ACS.LANGMUIR.6B03246/SUPPL_FILE/LA6B03246_SI_001.PDF

21.	SYLGARD^TM^ 527 A&B Silicone Dielectric Gel | Dow Inc. [cited 17 Feb 2025]. Available: https://www.dow.com/en-us/pdp.sylgard-527-ab-silicone-dielectric-gel.01512269z.html#overview

22.	Etezadi F, Tuyet Le MN, Shahsavarani H, Alipour A, Moazzezy N, Samani S, et al. Optimization of a PDMS-Based Cell Culture Substrate for High-Density Human-Induced Pluripotent Stem Cell Adhesion and Long-Term Differentiation into Cardiomyocytes under a Xeno-Free Condition. ACS Biomater Sci Eng. 2022;8: 2040–2052. doi:10.1021/ACSBIOMATERIALS.2C00162/SUPPL_FILE/AB2C00162_SI_007.MP4

23.	Deng Z, Wang W, Xu X, Nie Y, Liu Y, Gould OEC, et al. Biofunction of Polydopamine Coating in Stem Cell Culture. ACS Appl Mater Interfaces. 2021;13: 10748–10759. doi:10.1021/ACSAMI.0C22565/ASSET/IMAGES/LARGE/AM0C22565_0008.JPEG

24.	Chuah YJ, Koh YT, Lim K, Menon N V., Wu Y, Kang Y. Simple surface engineering of polydimethylsiloxane with polydopamine for stabilized mesenchymal stem cell adhesion and multipotency. Sci Rep. 2015;5: 18162. doi:10.1038/SREP18162

25.	Sharma D, Jia W, Long F, Pati S, Chen Q, Qyang Y, et al. Polydopamine and collagen coated micro-grated polydimethylsiloxane for human mesenchymal stem cell culture. Bioact Mater. 2019;4: 142–150. doi:10.1016/J.BIOACTMAT.2019.02.002

26.	Davidsen MB, Teixeira JFL, Dehli J, Karlsson C, Kraft D, Souza PPC, et al. Post-treatments of polydopamine coatings influence cellular response. Colloids Surf B Biointerfaces. 2021;207: 111972. doi:10.1016/J.COLSURFB.2021.111972

27.	Woo S, Chae R, Lee NY, Chae WR. Monolayer/spheroid co-culture of cells on a PDMS well plate mediated by selective polydopamine coating. J Mater Chem B. 2020;8: 10108–10116. doi:10.1039/D0TB01589J

28.	Yang DH, Jung S, Kim JY, Lee NY. Fabrication of a Cell-Friendly Poly(dimethylsiloxane) Culture Surface via Polydopamine Coating. Micromachines (Basel). 2022;13: 1122. doi:10.3390/MI13071122

29.	Sarkari S, Khajehmohammadi M, Davari N, Li D, Yu B. The effects of process parameters on polydopamine coatings employed in tissue engineering applications. Front Bioeng Biotechnol. 2022;10: 1005413. doi:10.3389/FBIOE.2022.1005413

30.	Moučka R, Sedlačík M, Osička J, Pata V. Mechanical properties of bulk Sylgard 184 and its extension with silicone oil. Scientific Reports 2021 11:1. 2021;11: 1–9. doi:10.1038/s41598-021-98694-2

31.	Johnston ID, McCluskey DK, Tan CKL, Tracey MC. Mechanical characterization of bulk Sylgard 184 for microfluidics and microengineering. Journal of Micromechanics and Microengineering. 2014;24: 035017. doi:10.1088/0960-1317/24/3/035017

32.	Masterton S, Ahearne M. Influence of polydimethylsiloxane substrate stiffness on corneal epithelial cells. R Soc Open Sci. 2019;6. doi:10.1098/RSOS.191796

33.	Dow. SYLGARD^TM^527 Silicone Dielectric Gel ® ^TM^Trademark of The Dow Chemical Company (“Dow”) or an affiliated company of Dow SYLGARD^TM^527 Silicone Dielectric Gel. 2017.

34.	Li X, Guo B, Xiao Y, Yuan T, Fan Y, Zhang X. Influences of the steam sterilization on the properties of calcium phosphate porous bioceramics. J Mater Sci Mater Med. 2016;27: 1–10. doi:10.1007/S10856-015-5617-X/TABLES/2

35.	Held P. Using Phenol Red to Assess pH in Tissue Culture Media.
